# Filling out the gaps – identification of fugralins as products of the *PKS2* cluster in *Fusarium graminearum*

**DOI:** 10.3389/ffunb.2023.1264366

**Published:** 2023-11-10

**Authors:** Manja Mølgaard Severinsen, Klaus Ringsborg Westphal, Mikael Terp, Trine Sørensen, Anders Olsen, Simone Bachleitner, Lena Studt-Reinhold, Reinhard Wimmer, Teis Esben Sondergaard, Jens Laurids Sørensen

**Affiliations:** ^1^ Department of Chemistry and Bioscience, Aalborg University, Aalborg, Denmark; ^2^ Institute of Microbiology and Microbial Biotechnology, University of Natural Resources and Life Sciences, Vienna, Austria; ^3^ Department of Chemistry and Bioscience, Aalborg University, Esbjerg, Denmark

**Keywords:** polyketides, secondary metabolites, natural products, PKSs, biosynthesis, pathogenicity

## Abstract

As one of the grain crop pathogenic fungi with the greatest impacts on agricultural economical as well as human health, an elaborate understanding of the life cycle and subsequent metabolome of *Fusarium graminearum* is of great interest. Throughout the lifetime of the fungus, it is known to produce a wide array of secondary metabolites, including polyketides. One of the *F. graminearum* polyketides which has remained a mystery until now has been elucidated in this work. Previously, it was suggested that the biosynthetic product of the *PKS2* gene cluster was involved in active mycelial growth, the exact mechanism, however, remained unclear. In our work, disruption and overexpression of the *PKS2* gene in *F. graminearum* enabled structural elucidation of a linear and a cyclic tetraketide with a double methyl group, named fugralin A and B, respectively. Further functional characterization showed that the compounds are not produced during infection, and that deletion and overexpression did not affect pathogenicity or visual growth. The compounds were shown to be volatile, which could point to possible functions that can be investigated further in future studies.

## 1 Introduction

*Fusarium graminearum* is known to be one of the plant pathogenic fungi with the largest impacts on agriculture worldwide, due to its infection of wheat, barley and corn (Dean et al., 2012). Not only, does the fungal infection cause up to 50% yield losses resulting in significant economic losses, it also leads to accumulation of mycotoxins causing severe health risks upon ingestion (Oadi, 2015; Friskop et al., 2021; Reed et al., 2022). These mycotoxins are typically classified as secondary metabolites, a group of biosynthetic products, which are typically produced to provide an advantage, thus supporting pathogens, such as *F. graminearum*, in an opportunistic lifestyle and complex infection strategy of vital crops (Chroumpi et al., 2020). Understanding of the secondary metabolism sheds light on the complex lifecycle of the pathogen, and thus presents possibilities for pathogen management. Additionally, several secondary metabolites including polyketides, have presented prospects as novel fungicides and antibiotics (Shude et al., 2020).

Polyketide synthases (PKSs), along with terpene synthases and nonribosomal peptide synthetases (NRPSs), are common core enzymes associated with secondary metabolism in multiple filamentous fungi, including those belonging to the genus *Fusarium* (Sieber et al., 2014; Hansen et al., 2015). Iterative type I PKSs are the only PKSs to be identified in *F. graminearum* (Malz et al., 2005; Dutta et al., 2014). PKSs are typically part of a biosynthetic gene cluster (BGC), where additional genes encode tailoring or transport enzymes, which are critical for the synthesis and regulation of the final product(s) (Brakhage, 2013; Nielsen et al., 2019). Great variety exist in the structure, complexity and function of polyketides produced by iterative type I PKSs (Herbst et al., 2018). Most fungal PKSs utilize acyl-CoA subunits as starter-units, which are extended and modified by a set of enzymatically active domains. The minimal domains of a PKS include a ketosynthase (KS) domain, an acyltransferase (AT) domain and an acyl carrier protein (ACP) domain and. The KS domain is responsible for intermediate elongation via a decarboxylative Claisen condensation, while the AT domain is responsible for recruiting elongation units, such as malonyl-CoA or acetyl-CoA. The ACP domain facilitates the transport between the active sites of each PKS domain (Till and Race, 2016; Herbst et al., 2018; Wang et al., 2020). Iterative type I PKSs are further classified as reducing or non-reducing, depending on which additional domains they contain. Reducing PKSs contain additional domains. the architecture typically starts with a KS- and an AT domain, followed by multiple reducing domains such as a dehydratase domain (DH), either a core domain or a methyl transferase domain (MT) or/and an enoyl reductase domain (ER), followed by a ketoreductase (KR) domain (Gaffoor et al., 2005; Dutta et al., 2014; Hansen et al., 2015; Herbst et al., 2018).

Ever since genome sequencing and subsequent genome mining revealed the presence of 15-16 *PKS* genes in the genome of *F. graminearum* (Gaffoor et al., 2005; Cuomo et al., 2007; Gardiner et al., 2014), much work has been put into elucidation of the structures and functions of the resulting polyketides (Keller, 2019; Poças-Fonseca et al., 2020). Many have been successful, and so far, 11 of the 15 PKSs typically found in *F. graminearum* have been linked to products, making it one of the best characterized polyketidomes (Nielsen et al., 2019). Despite the success regarding structural elucidation, the physiological roles of many of the polyketides remain unidentified.

One of *PKSs*, which have not yet been assigned to a product in *F. graminearum* is *PKS2*. The *PKS2* gene (FGSG_04694) was identified in 94 of the 204 analyzed species (Brown et al., 2022), including several prominent members of the *Fusarium* genus: *F. graminearum*, *F. pseudograminearum*, *F. verticillioides*, *F. fujikuroi*, *F. oxysporum*, *F. solani* and *F. meridionale* (Hansen et al., 2015; Walkowiak et al., 2016). PKS2 is a reducing type I PKS, which predicted contains seven domains: KS, AT, DH, methyltransferase (MT), ER, KR and ACP (Hansen et al., 2015). In the past, a serious growth impairment of a *F*. *graminearum* PH-1 *PKS2*-deletion mutant on carrot agar led to the conclusion that the metabolite is involved in the vegetative growth of the fungus, however, extensive transcriptomic analysis of the PH1 WT revealed no expression of *PKS2* during active vegetative growth on carrot agar after four, five and six days respectively (Gaffoor et al., 2005). *PKS2* expression was only observed during active growth in liquid YES (Gaffoor et al., 2005). Disruption of *PKS2* did not significantly affect pathogenicity on wheat, which was supported by transcriptomic analysis during infection of wheat (Gaffoor et al., 2005; Lysøe et al., 2011). The observed phenotype indicated that PKS2 might act as a regulator of mycelial proliferation.

In this work the role of the *PKS2* metabolic product was investigated throughout the entire lifecycle of the fungus through functional analysis of *Fusarium graminearum* PH-1 strains with *PKS2* (FGSG_04694) overexpressed or deleted respectively.

## 2 Materials and methods

### 2.1 Cluster analysis and bioinformatics

Amino acid sequences of KS domains from selected *Fusarium* PKSs were retrieved from previous studies (Hansen et al., 2012). Using CLC Main Workbench, the sequences were aligned and used to construct a Maximum likelihood phylogenic tree with 1000 bootstraps, which was visualized with EvolView (http://evolgenius.info/evolview). Synteny plots were generated using easyfig (Sullivan et al., 2011) based on the genome sequences of *F. fujikuroi*, *F. oxysporum*, *F. subglutinans*, *F. verticillioides*, *F. redolens*, *F. graminearum*, *F. pseudograminearum*, *F. vanettenii* and *F. duplospermum Penicillium griseofulvum* and *Daldinia* sp. The accession numbers and locus tags for the gene clusters are listed in **Supplementary File 1**.

### 2.2 Generation and verification of *PKS2* deletion and overexpression strains

Through the established USER friendly cloning technique designed for filamentous fungi (Frandsen et al., 2008) mutant strains were created from the *Fusarium graminearum* PH-1 (NRRL 31084) wild type (WT). The mutant strains were created using a targeted deletion and overexpression approach, which we have previously applied for successful generation of mutant strains with deletion- and overexpression of other *PKSs* in *F. graminearum* (*PKS6*, *PKS8, PKS9* and *PKS14*) (Sørensen et al., 2012; Jørgensen et al., 2014; Sørensen et al., 2014; Westphal et al., 2018). In brief, the deletion strain (*ΔPKS2*) was created by targeted replacement of the *PKS2* gene with the hygromycin resistance cassette via homologous recombination. The overexpression (*OE::PKS2*) of *PKS2* was facilitated by insertion of the constitutive PgpdA promoter element in front of the *PKS2* gene (FGSG_04694) and the hygromycin resistance cassette in locus. The resulting transformants were identified by diagnostic colony PCR and full genome sequencing (**Supplementary Figure 1**). DNA extraction and purification for sequencing were performed via the phenol-chloroform method according to the protocol previously described (Petersen et al., 2022). The coverage for the sequencing was 200 for the overexpression mutant and 100 for the deletion mutant. The primers used for mutant construction and validation are listed in **Supplementary File 1**.

### 2.3 Metabolite analyses

In our endeavors to identify the resulting products of the *PKS2* cluster, we initially analyzed the volatile metabolome of the strains, which were collected from cultures cultivated in 20 mL glass vials containing 3.33 mL YES agar medium for seven days at 25°C. After cultivation, the vials were placed at 50°C for 30 minutes, where the volatile metabolites were extracted by head space solid phase microextraction (HS-SPME) using a d_f_ 65 μm polydimethylsiloxane/Divinylbenzene (PDMS/DVB) fiber. The extracted volatile metabolites were injected into a GC-MS (Perkin-Elmer) using a gradient starting at 40°C for one minute and then increased to 200°C in 16 minutes, which was held for 4 minutes. After each analysis, the fiber was cleaned while being in the GC-MS at 225°C for 10 minutes.

For further analyses, the strains were grown in 90 mm plates with YES agar medium at 25°C in the dark for 14 days. Then we used an extraction protocol inspired by Smedsgaard, 1997 and Westphal et al., 2021. 2 mL ethylacetate:dichloromethane:methanol (3:2:1) with 1% formic acid was added to the grounded material followed by 5 min sonication at room temperature. The solvent was decanted into fresh tubes and subjected to a flow of N_2_ gas to allow evaporation of the solvent. The metabolites were then resuspended in 200 µL methanol and precipitated materials were pelleted by centrifugation for 5 min, 14.000 rpm, the supernatant was then transferred to HPLC-vials. 20 µL of the respective extract was injected into the Phenyl-Hexyl column (Ascentis^®^ Express, Phenyl-Hexyl 150 × 4.6 mm, 2.7 µm pore size, Sigma-Aldrich St. Louis, MO, USA), the column was kept at 40°C on a Hitachi Elite LaChrom HPLC system (Hitachi Ltd., L-2130 pump, L-2200 autosampler, L-2300 oven, L-2450 DAD detector, Tokyo, Japan). The DAD detector recorded absorptions between 190-900 nm. I HPLC system was coupled with a High-Resolution Mass Spectrometer (HRMS; Bruker Compact MS ESI-qTOF, Bruker Daltonics, Bremen, Germany) using a 5:95 flowsplitter. The electron spray ionization source was operated in positive mode (4500 V (capillary), 500 V (end plate off-set), 4.0 L-min-1 dry gas. 200°C). For chromatographic separation solvent A (LCMS-grade acetonitrile with 0.1% formic acid) and solvent B (LCMS-grade water with 0.1% formic acid) was applied to the column with a flow rate of 1.2 mL.min-1. Following 4 min prerun, a gradient was initiated at 10% solvent A and was linearly increased to 100% over the next 10 min, where it was maintained for 20 min. For calibration, 2.0 µL HRMS calibrant was injected into the mass spectrometer after 0.2 min. Between different sample types/triplicates, the HPLC column was washed using a standard washing program. The HPLC-DAD-HRMS setup was controlled with HyStar v3.2. Data analysis was conducted with Bruker Compass Data Analysis v4.2 Software, which was also used for prediction of the chemical formulas of the target compounds.

### 2.4 Isolation and structural elucidation

The OE::*PKS2* strain was grown on 30 YES plates at 25°C in the dark for 10 days. The plates were extracted with 1.4 L ethyl acetate (containing 1% formic acid) in an ultrasonic bath for 45 minutes. The extract was filtered through Mira-cloth and evaporated to dryness under a stream of N_2_. The sample was redissolved in 2 mL methanol and centrifuged for 2 minutes at 12.000 rpm. The supernatants were analyzed by HPLC-MS-SPE-NMR performed on a system consisting of a Hitachi Elite LaChrom HPLC equipped with a L-2130 pump, a thermostated L-2300 column oven (40°C), a column (Ascentis Express RP-amid, 15 cm × 4.6 cm, 2.7 μm particle size), a L-2200 autosampler and a L-2400 UV detector; a Bruker “compact” mass spectrometer (Bruker Daltonics) equipped with an electrospray ionization source and a 3:97 flow splitter; a BNMI makeup pump (VICI, Valco Instruments Co. Inc.); a Knaur Smartline K120 pump (Knaur) for dilution prior to trapping, a Spark Holland Prospekt 2 SPE unit (Spark Holland); a liquid handler (Gilson F215). The instrument was controlled with Hystar 3.2 (Bruker Daltonics) and the data was processed with Compass^®^ DataAnalysis 4.2 (Bruker Daltonics). Injection volume was 40 μL. A gradient of water with 0.1% (v/v) formic acid (Fisher Scientific) together with acetonitrile with 0.1% formic acid was used with a flow of 1 mL/minute. The gradient was initiated with 10% acetonitrile, which was increase linearly to 99% acetonitrile over 20 minutes. 99% acetonitrile was kept until 25 minutes. Between runs a pre-run phase was conducted for 5 minutes with 10% acetonitrile.

Two selected extracted ion chromatogram peaks (trapping–1 - 2) from the mass spectrum were trapped with 46 repetitive separations on individual 2 mm i.d. Resin GP (general purpose,–5 - 15 μm, spherical shape, poly(divinylbenzene) phase) SPE cartridges (Spark Holland) with a makeup flow (K120) of 2 mL/minute for peaks–1 - 2, 1 mL/minute. Subsequently the cartridges were dried for 1 hour with a stream of nitrogen.

The isolated compounds were dissolved in methanol-d_4_ and structurally elucidated by nuclear magnetic resonance spectroscopy using a BRUKER AVIII-600 MHz NMR spectrometer equipped with a CPP-TCI cryogenically cooled probe (Westphal et al., 2019).

### 2.5 Reproductive cycles

Conidiation of macroconidia for morphology testing, fungal growth tests and pathogenicity assays was performed in mung bean soup (MBS). MBS is prepared by boiling 10 mung beans for 20 min followed by filtering through two layers of Mira-cloth. 50 mL of MBS in 250 mL baffled flasks were inoculated with two 5 mm round agar plugs with vegetative mycelium of the respective strains, and cultures were shaken at 140 rpm for three days, at 20°C in the dark. Macroconidia were collected by filtering the inoculated media through glass wool or two layers of Mira-cloth into a 50 mL centrifuge tube. The tube was then centrifuged for 30 min, 2000 rpm, and the supernatant was carefully discarded. Macroconidium solutions were diluted, and the morphologies of the respective macroconidia were manually assessed through microscopic analyses. Calcofluor-white staining was performed by adding 10 µL spore-dilution to a glass slide, followed by 5 µL Calcofluor-white and 5 µL 10% potassium hydroxide. Stained and unstained macroconidia were imaged using an Olympus IX83 inverted microscope with a Yokogawa CSU-W1 spinning disk unit equipped with a Hamamatsu Orca-Flash 4.0 camera using 100X. Z-stacks were acquired and maximum projections over Z images we generated using the Olympus CellSens software. The macroconidia were analyzed using differential interference contrast microscopy and a DAPI emission filter (365 nm excitation:440 nm emission) Ascospores were produced as described by Cavinder et al., 2012. In detail, freshly prepared carrot agar plates were center inoculated with one mycelium-covered agar plug of the respective strains. The plates were sealed with parafilm and incubated until the agar was fully covered with mycelium. The incubation conditions were at room temperature and -humidity in a windowsill or in an incubator with controlled light/dark conditions (light: 16 h, dark: 6 h). When the plates were fully covered with mycelium, the mycelium was scraped off and disrupted with a sterile cotton stick, 1 mL 2.5% Tween 60 was applied and dispersed on the plates, and the plates were reincubated, this procedure was repeated every other day until the plates were covered with perithecia (~3 weeks). Ascospores were extracted by mashing lumps of perithecia between two glass slides followed by a dilution in sterile water. The presence and morphology of the ascospores was investigated microscopically,

Using the live-cell imaging system, oCelloScope™ (BioSense Solutions), we performed an automated germination test. The germination process of macroconidia and ascospores was investigated in four replicates in different liquid media: sterile water, Potato Dextrose Broth (PDB), Yeast Peptone and Glucose (YPG) and MBS. 200 µL of the respective media containing a total of 5000 spores was transferred to each well of a 96 deep well plate. The plate was incubated at 20°C in the oCelloScope™ and the germination process was recorded over a period of two days. The obtained images were used for generation of growth curves by using the highly sensitive image-based normalized Background Corrected Absorption (BCA) algorithm. Morphological changes and germination of asexual and sexual spores evaluated manually from the recorded images.

### 2.6 Vegetative growth

For analysis of the vegetative growth under different nutritional conditions, either 1000 macroconidia or an agar plug covered in mycelium of the respective *F. graminearum* strains, i.e., WT, Δ*PKS2*, and OE::*PKS2*, were inoculated on an array of media at 20°C in the dark. Solid complete media (CM; Pontecorvo et al., 1953) and Yeast Extract with Supplements (YES; (Sørensen and Søndergaard, 2014)) were used to investigate saprophytic growth under optimal conditions, while potato dextrose agar (PDA; Roth) was used to investigate growth on a natural plant based medium, synthetic Imperial Chemical Industries, Ltd, UK media (ICI; Geissman et al., 1966) supplemented with 6 mM glutamine as the only nitrogen source. To test sensitivity to oxidative or osmotic stress, CM was supplemented with either 10 mM H_2_O_2_ (30% H_2_O_2_, Roth), 0.5 mM menadione sodium bisulfite (Sigma-Aldrich), 1 M sorbitol (Sigma-Aldrich) or 1 M sodium chloride (Carl Roth), respectively. Inoculation with 1000 macroconidia allowed comparison of the initiation of vegetative growth following germination, while inoculation with an agar plug covered with mycelium of the respective strain was performed to exclude the effects of collapsed or non-functional macroconidia. All experiments were conducted in triplicate. Radial growth was monitored over a period of up to 7 days of incubation at 20°C in the dark. Average radial growth and standard deviations were calculated based on two measurements of the radial diameter of each mycelium performed on each set of triplicates.

### 2.7 Pathogenicity testing

Infection experiments were carried out as described elsewhere (Studt et al., 2017). Briefly, highly susceptible USU-Apogee full-dwarf hard red spring wheat (*Triticum aestivum* cv. USU-Apogee; Reg. no. CV-840, PI592742) was utilized to investigate the virulence of the different *F. graminearum* strains. Two viable, flowering wheat ears were infected in two neighboring spikelets per wheat ear.Each spikelet was infected by pipetting a water suspension containing 1000 spores of the respective strain under the glume into the blooming caryopsis. Sterile water was used as inoculum to simulate a mock infection. Five replicas or more were made during each experiment. To promote fungal infection newly infected ears were covered with a plastic bag with 3 mL of sterile water for 24 h after initial infection. Infection-spreading between flanking wheat spikelets was observed with alternating time intervals. The wheat ears were then harvested and frozen in liquid nitrogen followed by cryodesiccation and analysis to elucidate the secondary metabolite profile.

The secondary metabolite profile of the dry infected wheat ears was analyzed following secondary metabolite extraction of triplicates. Approximately 80 mg of ground wheat heads, fungal-infected and water-infected, were used for extraction.

## 3 Results

### 3.1 Zooming in on the *PKS2* gene cluster

**Figure 1:**
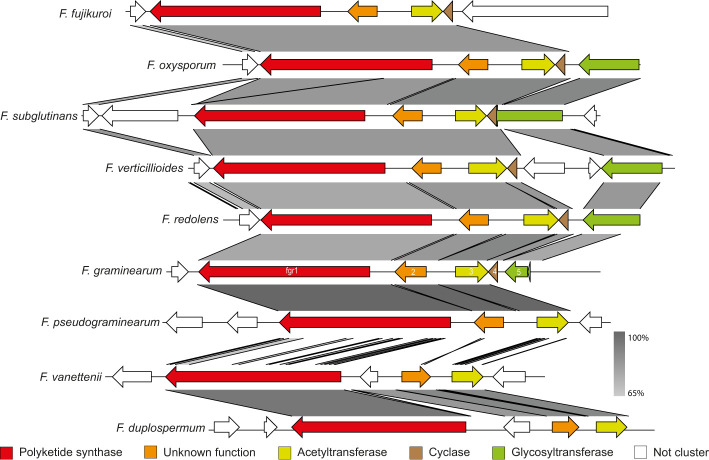
Synteny plot of the PKS2 cluster in *F. fujikuroi, F. oxysporum, F. subglutinans, F. verticillioides, F. redolens, F. graminearum, F. pseudograminearum, F. vanettenii and F. duplospermum.* Color codes have been assigned to the five potential members of the gene cluster, while non-cluster genes are in white. The predicted function of the genes was determined through the NCBI Conserved Domain Database.

To determine the members and boundaries of the *PKS2* cluster, we initially constructed a synteny plot of nine *Fusarium* species. The analyses showed that four genes next to *PKS2* in *F. graminearum* were also present in *F. verticillioides* as previously observed (**Figure 1**; (Zhao et al., 2014). Besides *PKS2* (*fgr1*), these genes are predicted to encode a protein with unknown function (*fgr2*; FGSG_04695), an acetyltransferase (*fgr3*; FGSG_04696), a cyclase (fgr4; FGSG_12583) and glycosyltransferase (*fgr5*; FGSG_12582 + 1). These five genes were also present in *F. oxysporum* and *F. subglutinans*, although *fgr4* and *5* are annotated as one gene in the latter species. However, *fgr5* is absent in *F. fujikuroi*, despite being closely related to *F. verticillioides* and *F. subglutinans*, which suggests that fgr5 may not be part of the cluster. This is further emphasized in a transcriptomic analysis, where only fgr1-4 were co-expressed in *F. verticillioides* (Butchko et al., 2012). Interestingly, the predicted cyclase was absent in *F. pseudograminearum*, despite *F. graminearum* and *F. pseudograminearum* being closely related and the gene clusters share a high sequence similarity (Walkowiak et al., 2016). In the more distantly related *F. redolens* and the two members of the *F. solani* species complex, *F. vanettenii* and *F. duplospermum*, only three orthologues (*fgr1-3*) were identified, which could further suggest that the gene cluster only consists of these three genes. This is also supported by a study in *F. verticillioides*, where deletion of *SGE1*, which is a transcriptional regulator of transitional development, significantly reduced transcription of *fgr1-4*, while *fgr5* was not affected (Brown et al., 2014).

In a large genus-wide analysis of PKSs in *Fusarium*, PKS2 was placed in the reducing PKS clade I (Brown et al., 2022). Of the PKSs with known products in *F. graminearum*, this clade also includes PKS6 (fusaristatin; Sørensen et al., 2014) and PKS4 (zearalenone; Kim et al., 2005; Lysøe et al., 2006). In the reducing clade I, PKS2 found to be closely related to PksF from *Alternaria solani* (Wiemann et al., 2013; Brown et al., 2022), which is responsible for biosynthesis of aslanipyrone (Kasahara et al., 2006). This therefore suggested that the product of PKS2 could be a highly reduced molecule, potentially a pyrone similar to aslanipyrone.

### 3.2 Identification of fugralins as products of the *PKS2* gene cluster

**Figure 2:**
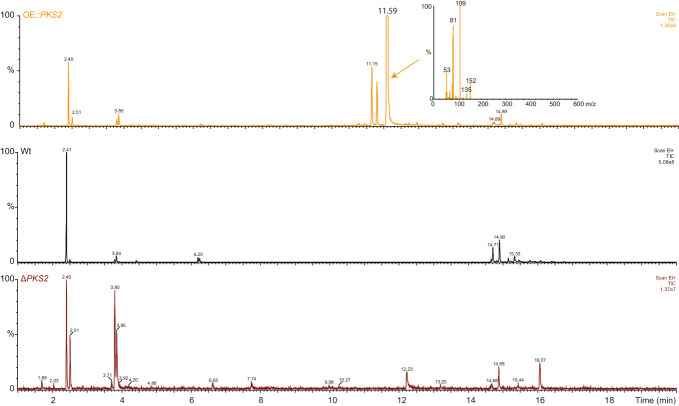
Analyses of the volatile metabolites produced by the *F. graminearum* Wt, OE::*PKS2* and Δ*PKS2* by SPME-GC-MS. The mass spectrum of the high unique peak in OE::*PKS2* has been inserted.

Based on the phylogenetic analyses, we theorized that the products of the PKS2 cluster could be volatile, and a HS-SPME-GC/MS was therefore applied to analyze the metabolome of the WT, OE::*PKS2* and Δ*PKS2* strains (**Figure 2**). The resulting chromatograms showed that three clear peaks were present in the OE::*PKS2* strain, which were absent in the WT and Δ*PKS2* strains. In the largest peak, the mass spectrum showed fragment with *m*/*z* values of 152, 135, 109, 81 and 53 as the most abundant fragments.

**Figure 3:**
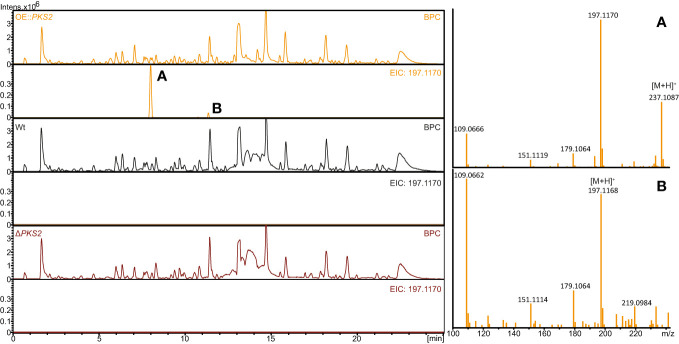
HPLC-HRMS analyses of secondary metabolites produced on solid YES plates in the *F*. *graminearum* Wt, OE::*PKS2* and Δ*PKS2*. The base peak chromatograms (BCP) and extracted ion chromatograms (197.1170 ± 0.02) are depicted for each strain, while the mass spectrum for the two isolated compounds (fugralin **(A, B)** and shown for the OE::*PKS2* strain.

Subsequently, comparison of metabolite extracts from the three strains analyzed with LC-HRMS showed similar results with two compounds (A; [M+H]^+^: 237.1087 and B; [M+H]^+^:197.1168) only present in the OE::*PKS2* strain (**Figure 3**). Both compounds showed fragment with *m/z* values of 197, 179, 151 and 109. Further analysis suggested two different chemical formulas for compounds A (C_11_H_18_O_4_) and B (C_11_H_16_O_3_) with retention times at 8.0 and 11.2 minutes, respectively.

**Figure 4:**
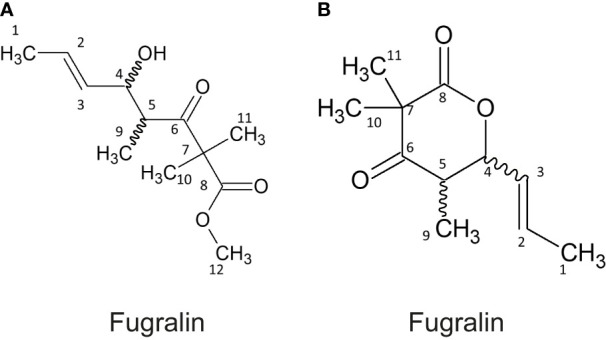
Structures of fugralin **(A, B)**.

These compounds were targeted for isolation by preparative HPLC-MS and subsequently elucidated by 1D and 2D NMR experiments. During purification, we experienced that the mass of compound A increased by 14 g/mol, corresponding to addition of a methyl group, which is most likely derived from the methanol used in the extraction process. The correlated spectroscopy (COSY) spectra revealed a spin system initiating at H^1^ through to H^5^ and ending at H^9^, for both compound A and B. The remaining topology of both compounds were established by Heteronuclear Multiple Bond Correlation (HMBC) (**Table 1**), establishing that compound A, which we named fugralin A, is a linear partly reduced tetraketide with a double methyl group (C^10-11^) on C^7^, ending with a carboxymethyl at C^12^ (**Figure 4**). We propose that the carbomethyl group is derived from a reaction during the purification and that the naturally produced molecule contains a carbolic acid group on C^8^. The trans configuration of the doublebond between C2 and C3 was determined by a coupling at 15.1 Hz. Fugralin B (compound B) was similar to fugralin A except for a cyclization between the carboxylic acid C^8^ and the alcohol on C^4^ resulting in a six membered lactone ring (**Figure 4**). The absolute stereochemical configuration of the two stereocenters (C^4^ and C^5^) of fugralin A and B could not be resolved.

**Table 1:** NMR spectroscopic data (600 MHz, MeOD-d_4_, 298.1 K) of fugralin A and B.

	Fugralin A				Fugralin B			
#	δ_C_	Type	δ_H_ (J in Hz)	HMBC	δ_C_	Type	δ_H_ (J in Hz)	HMBC
1	17.8	CH_3_	1.71 (d, 6.5)	2,3,4,5	18.1	CH_3_	1.79 (d, 6.6)	2,3
2	129.8	CH	5.68 (dq, 6.5, 15.1)	1,4	134.6	CH	5.95 (dq, 6.6, 15.1)	1,4
3	133.1	CH	5.4 (dd, 8.1, 15.1)	1	129.1	CH	5.55 (dd, 8.4, 15.1)	1
4	76.7	CH	4.01 (dd, 8.1, 9.0)	2,3,5,9	82	CH	4.74 (dd, 8.4, 11.0)	2,3,6,9
5	48.5	CH	2.94 (dq, 6.9, 9.0)	3,4,6,9	46.9	CH	2.73 (dq, 6.8, 11.0)	3,4,6,9
6	213.4	C			209.9	C		
7	57.8	C			53	C		
8	175.2	C			177	C		
9	16.1	CH_3_	0.9 (d, 6.9)	4,5,6	11	CH_3_	1.04 (d, 6.8)	4,5,6
10	22.4	CH_3_	1.35 (s)	6,7,8,11	24	CH_3_	1.37 (s)	6,7,8,11
11	22.2	CH_3_	1.4 (s)	6,7,8,10	24.1	CH_3_	1.39 (s)	6,7,8,10
12	52.7	CH_3_	3.69 (s)	7,8				

**Figure 5:**
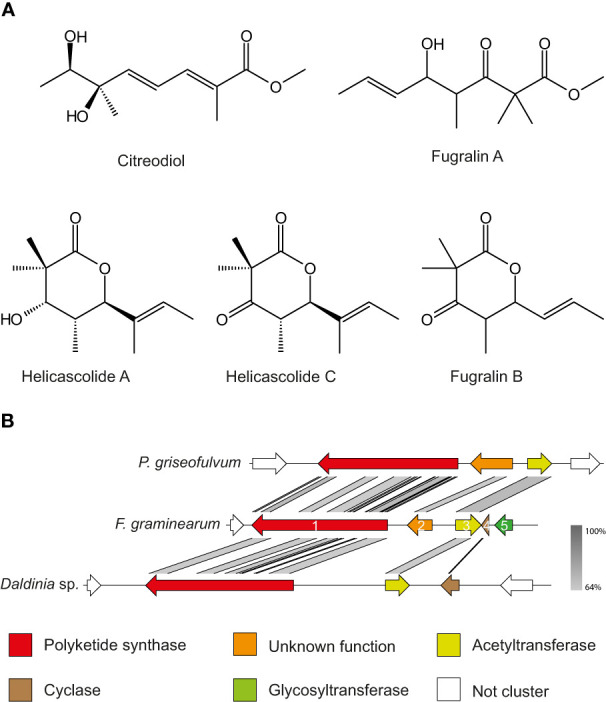
**(A)** Structures of fugralins and the related citrodiol and helicascolide A and C Stereochemistry of fugralins was not resolvable and therefore not included. **(B)** Synteny plot of the *PKS2* cluster in *Penicillium griseofulvum*, *F*. *graminearum* and *Daldinia* sp. Color codes have been assigned to the five members of the gene cluster, while non-cluster genes are in white.

One of the key features of fugralins is the presence of a double methyl group, which is only rarely encountered in fungal secondary metabolites. Searching the literature for double methylated polyketides, we found two compounds from *Daldinia eschscholzii* named helicascolide A and C, which share remarkable similarity to fugralin B (Tarman et al., 2012) with the only difference being the presence of an extra methyl group on C2 (**Figure 5A**). As the *PKS2* cluster does not contain an independent methyltransferase, we predict that the PKS is responsible for adding two methyl groups to the same carbon atom. Similarly, fugralin A shares some similarity to citreodiol, which has been isolated from *Penicillium citreoviride* (Shizuri et al., 1984). This compound has a methoxy group and a carbon atom with two functional groups (methyl and hydroxyl). This illustrates that the ability to produce compounds similar to fugralins is also found outside the *Fusarium* genus.

In our study we have shown that fugralins are produced when *PKS2* is overexpressed. However, the role of the individual cluster genes remains unknown and further work is needed to unravel the biosynthetic pathway. Interestingly, we have identified a gene cluster in a *Daldina* sp., which contain orthologues of *PKS2* as well as the acetyltransferase *fgr3* and the cyclase *fgr4* (**Figure 5B**). Since the cyclic helicascolide A and C were discovered in *D. eschscholzii*, this suggests that fgr4 could be involved in a cyclization reaction. This is further supported by another orthologous cluster in *Penicillium griseofulvum* (a close relative of P. citreoviride), which contain orthologues of *PKS2* as well as *fgr2* and *fgr3*. The cluster does not contain an orthologue of *fsr4*, which corresponds to only linear related compounds reported from *F. citreoviride*

### 3.3 *PKS2* expression affects macroconidia morphology but not ability to germinate

Macroconidia were produced by all strains after two to three days when cultivated in liquid MBS. Real-time analysis demonstrated that the macroconidia of the mutant strains were able to germinate and showed WT-like germination patterns in all tested media. The growth curves generated showed similar growth patterns of the *F. graminearum* WT and the mutant strains (data not shown). During macroconidia quantification, it was observed that macroconidia of both mutant strains appeared different from the WT macroconidia, which is why additional microscopic analysis at higher resolution and with Calcofluor-white staining of the fungal cell walls was performed.

**Figure 6:**
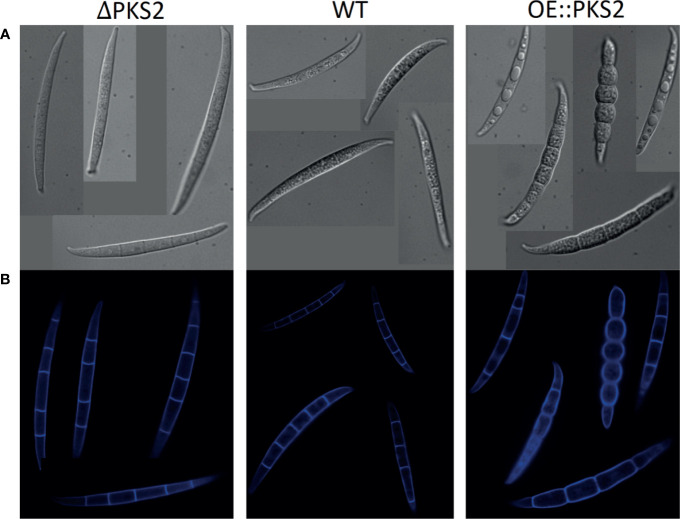
Macroconidia of the three respective *F. graminearum* strains: PKS2Δ, WT and OE::PKS2 dyed with Calcuflour white staining. **(A)** Images from DIC microscopy. **(B)** Images from microscopy with a DAPI emission filter.

From DIC microscopy (**Figure 6A**) the phenotypes of the deletion and overexpression strain macroconidia clearly differed from the WT-macroconidia. The macroconidia of Δ*PKS2* appear slightly smoother and more transparent than those of the WT, while the OE::*PKS2* macroconidia all appear swollen. For some OE::*PKS2* macroconidia the swelling is less excessive, however, vesicles of unknown composition appear within each septate cell. When analyzing the macroconidia with a DAPI emission filter (**Figure 6B**), the Calcofluor-white staining reveals that all macroconidia contain septa.

Perithecia formation on carrot agar was observed for the overexpression and deletion strain in a similar manner to the WT, the duration for formation and morphology of the perithecia were WT-like (data not shown). The perithecia of both mutant strains as well as the WT produced ascospore which were able to germinate in an array of media. Continuous real-time analysis using oCelloScope revealed that germination initiation and progression were similar for all strains.

Through the real-time analysis of the germination process of ascospores and initiation of vegetative growth of ascospores, an interesting phenomenon was observed for all strains. In all investigated media (sterile water, PDB, YPG and MBS) the newly formed germtubes emerging from ascospores would initiate macroconidia production. The same phenomenon was not observed under the same conditions when the germtubes emerged from germinating macroconidia, thus suggesting large differences in the germination processes and resources of *F. graminearum* macroconidia and ascospores.

### 3.4 Manipulation of *PKS2* has only minor effect on growth

To investigate whether the mutant strains deviated from the WT during vegetative growth an array of media with different nutritional conditions were chosen: ICI, CM, PDA and YES. CM supplemented with either hydrogen peroxide, menadione, sorbitol or sodium chloride were chosen to investigate whether fugralin biosynthesis is involved in either oxidative or osmotic stress response.

**Figure 7:**
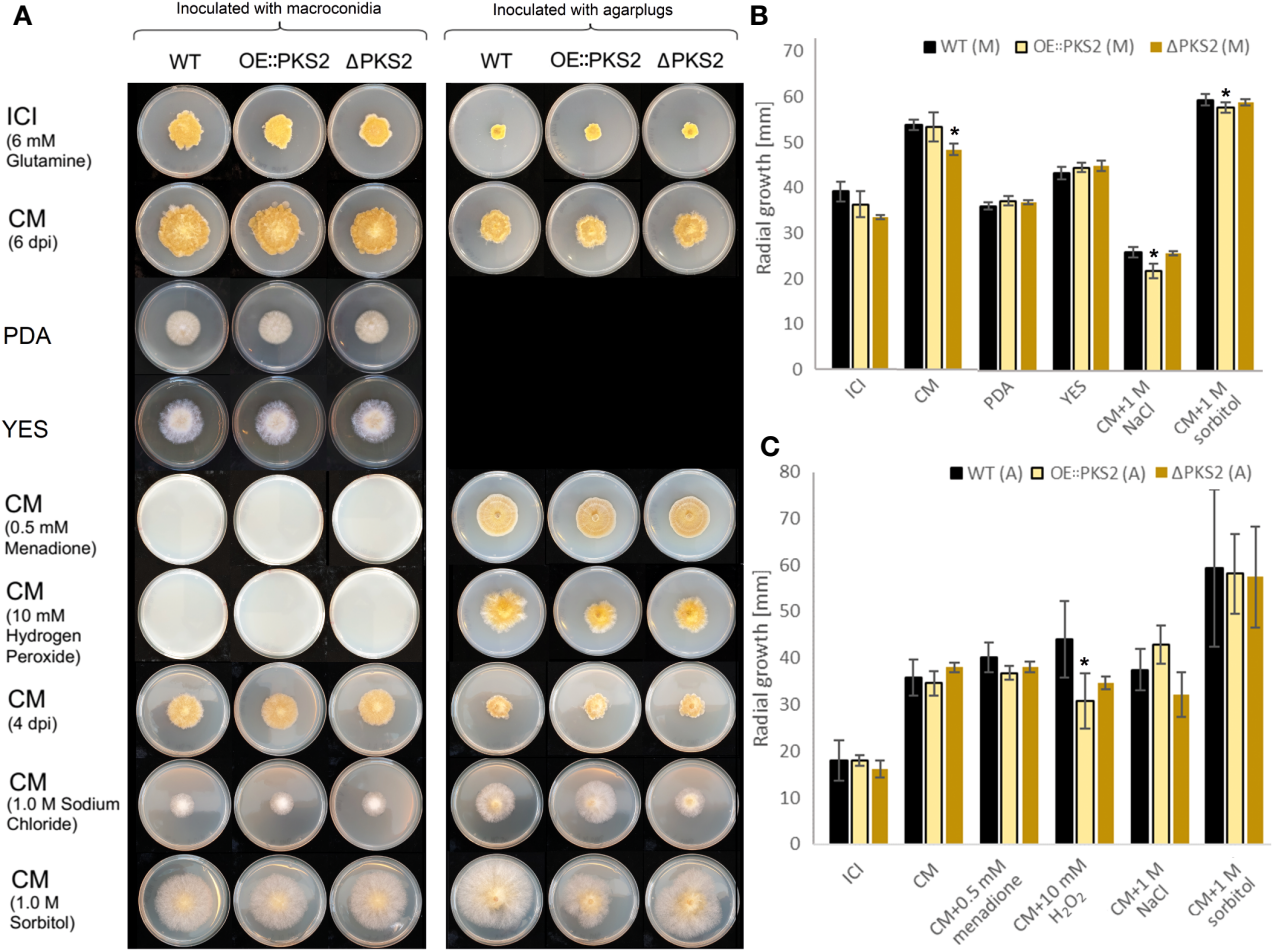
Radial growth test of the *F. graminearum* WT, OE::*PKS2* and Δ*PKS2*, inoculated on different media. The experiments were performed in triplicates. **(A)** Visalization of the radial growth of the fungal strains inoculated on an array of media with 10000 macroconidia (left) or one agar plug (right). Images were taken at six dpi on ICI, CM, and CM supplemented with respectively menadione and hydrogen peroxide. Images of the growth on CM, PDA and YES and CM supplemented with either sorbitol or sodium chloride were taken at four dpi. **(B)** The radial growth on ICI and CM was measured at six dpi with macroconidia (M), while the radial growth was measured at 4 dpi with macroconidia for PDA, YES and CM supplemented with sorbitol or sodium chloride. A Students T-test was performed to investigate stitistical signficance in the radial growth of the PKS2 mutants respective to the WT, *:p<0.05. **(C)** The radial growth on ICI, CM and CM supplemented with menadione or hydrogen peroxide was measured at six dpi with agar plugs **(A)**, while the radial growth was measured at four dpi with agar plugs for CM with sorbitol or sodium chloride **(A)**.

The morphology of the vegetative mycelium of the two mutants on each of the respective media were similar to the WT (**Figure 7**). On all media, except PDA and CM supplemented with sorbitol, yellow pigmentation of the mycelium of all strains was observed. Macroconidia of all strains were unable to germinate on CM supplemented with the oxidative stressors, menadione and hydrogen peroxide, while vegetative growth was possible for all strains. The growth on CM with menadione was very symmetrical, while the fungal spreading on CM supplemented with hydrogen peroxide appeared uneven, suggesting that the two oxidative stressors affect fungal growth through different mechanisms. On osmotic stress media, CM supplemented with sodium chloride or sorbitol, the production of aerial mycelium was increased while the mycelium density appeared to be decreased for all strains, although large discrepancies were observed between fungal growth on the two media. The supplementation with sorbitol increased the fungal growth rate of all strains following inoculation with agar plugs as well as with macroconidia. On CM supplemented with sodium chloride, the difference in radial spread between the two inoculation methods indicate that the germination of all strains was negatively affected by the osmotic stress.

### 3.5 Virulence assay indicates no significant effect of Fugralins on virulence

**Figure 8:**
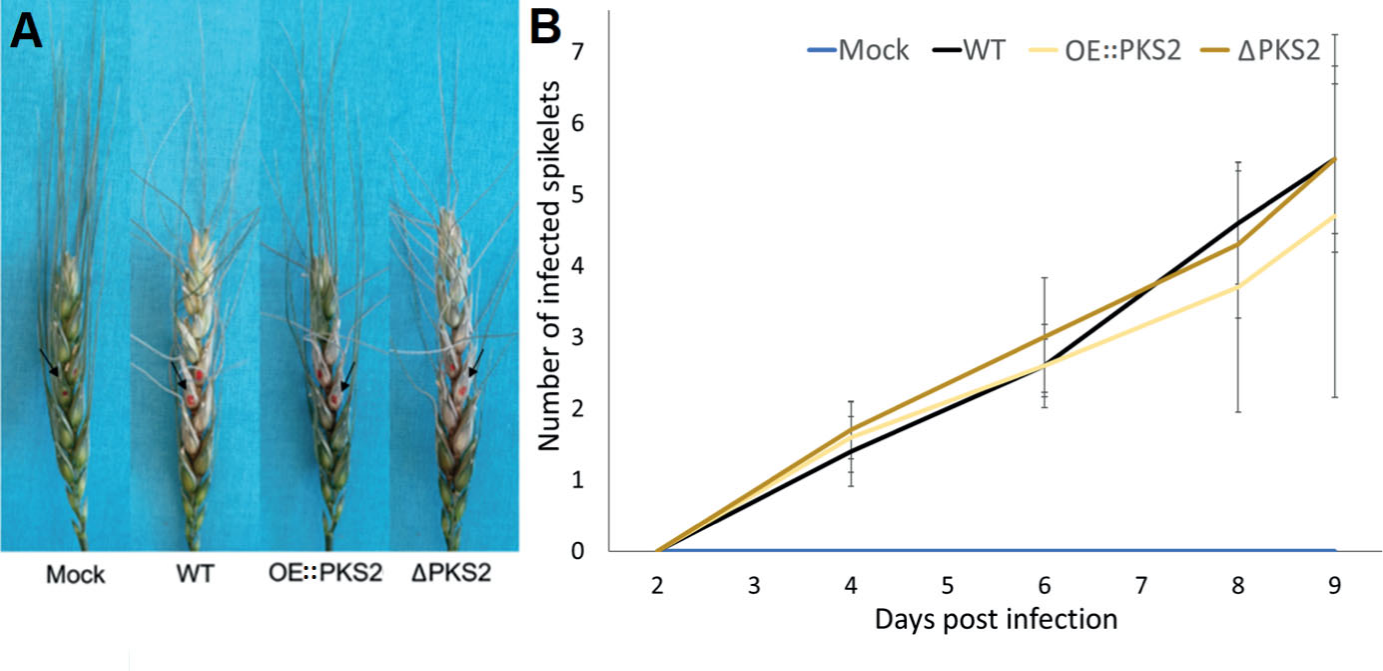
Wheat infection assay using macroconidia from WT, OE::*PKS2* or Δ*PKS2* and with water (mock) as control. **(A)** Harvested, infected wheat ears at 12 dpi with the inoculated spikelets are marked with red. **(B)** The average infection spreading over time, mean values and standard deviations are depicted.

To determine whether fugralins are involved in pathogenicity, highly Fusarium Head Blight (FHB) susceptible (Mackintosh et al., 2006) APOGEE-wheat ears were infected with macroconidia of the WT, Δ*PKS2* and OE::*PKS2* respectively. The characteristic symptoms of FHB were observed on wheat ears infected with the WT and *PKS2* mutants, no symptoms were observed for mock-inoculated wheat ears (**Figure 8A**). For all strains, infection of flanking wheat spikelets was possible from infected spikelets. The infection of each respective strain has large standard deviations, which is why neither disruption nor overexpression of *PKS2* significantly affected fungal spreading throughout the wheat ear. The results of **Figure 8B** depict a single virulence assay out of three performed.

In the subsequent metabolic analyses, fugralins could not be detected in any of the samples, which collectively suggests that they are not involved in pathogenicity. To improve the accuracy of the virulence assay, alternative inoculation methods with direct injection into the stalk at the susceptible caryopsis, instead of pipetting the spores on the exterior of the caryopsis. This avoids the bias of the wheat susceptibility, that is determined by the developmental stage of the individual wheat ear, resulting in large standard deviations. Additionally, more replicas could be included. Under environmental conditions, fungal spores would have multiple opportunities to initiate an infection.

Although the results of the virulence assays are statistically insignificant due to large standard deviations, the same patterns of infection, that OE::*PKS2* seems to have a slower infection spread than the WT and the Δ*PKS2*, were observed in three independent virulence assays. The observable infection of the OE::*PKS2* mutant on average was only 28% less than for the WT, while it for the Δ*PKS2* mutant was 8% higher throughout the supervised periods.

## 4 Discussion

PKS2 is found in in nearly half (94/206) of the genome sequenced *Fusarium* species, which makes it one of the most conserved PKSs in the genus (Brown et al., 2022). The available transcription data provides little clue to the role of *PKS2*, as it is only rarely transcribed in *F. graminearum*. Our results from the growth experiments and pathogenicity trials suggested that fugralins are not essential roles in these processes. The phenotypical differences between the mutant macroconidia and the WT suggest some involvement of fugralins in the asexual lifecycle of the fungus, however, as no differences in germination ability nor growth were found, the function of fugralins remains unresolved. In a screen for bioactivity, helicascolide C showed fungistatic effects against *Cladosoporium cucumerinum*, while both did not exhibit antibacterial effects against *Escherichia coli, Pseudomonas aeruginosa, Staphylococcus aureus* or *Bacillus subtilis* (Tarman et al., 2012).

PKS7 is the most conserved (201/206) and although it has not yet been assigned to a product, deletion studies have indicated a role in sexual development resulting in abnormal conidiation (Kim et al., 2015). This is intriguing since, deletion of overexpression of *PKS2* was also found to affect conidia morphology in our study. This effect could be mediated by the volatile nature of the isolated fugralins, as suggested by the SPME-GC-MS analyses. Volatile compounds (VOCs) can have plethora of different functions and each VOC can have different functions at varying concentrations and against different species (Poveda, 2021). They are commonly used to signal other microbes in the same kingdom either to establish a symbiotic relationship or contribute to pathogenicity (Weisskopf et al., 2021). VOCs can also be used as intra-kingdom signals, where fungi can signal bacteria, bacteria can signal fungi and they can both signal mammals, plants and insects. Only in the past decade has VOC research gained traction (Schulz and Dickschat, 2007; Weisskopf et al., 2021). Due to the transient nature of the metabolites and the spatiotemporal gene expression of the *F. graminearum* throughout its lifecycle, the purification and therefore structure elucidation of specific VOCs can be difficult, and further analysis of the biological activity of fugralins was not possible.

The functional analysis of the OE::*PKS2* and Δ*PKS2* mutants did not confirm the previous conclusions that *PKS2* is involved in growth and/or virulence (Gaffoor et al., 2005; Lysøe et al., 2011), as no significant differences in growth on the selected array of media, nor on the virulence toward wheat in the demonstrated assays were observed. However, clear differences in the morphology of the macroconidia of the mutant strains and the WT indicate that fugralins play a role in the asexual reproductive cycle, which is indisputably related to vegetative growth as well as virulence. Additional experiments, looking into germination and conidiation ability on carrot agar, the macronidial morphology as well as germination process would make a valuable contribution to elucidate the exact function of the metabolite. There is a possibility that overexpressing the entire cluster and not only *PKS2*, could lead to more significant effects, which will have to be investigated in the future.

In our study we have identified the fugralins through overexpression of the PKS and is therefore possible that the isolated compounds are not the end product of the biosynthetic pathway. This would only be the case if the remaining genes are also expressed. Future studies will have to clarify this. Despite the yet unknown function of fugralins, this study moves one step closer to revealing the entire polyketideome. 12 of the 15 *PKSs* in *F. graminearum* have now been linked to a product (Nielsen et al., 2019), which leaves only *PKS1*, *PKS5* and *PKS7* with unknown products. This places *F. graminearum* as one of the best characterized fungal species in terms of known polyketide pathways, which in the future can provide valuable knowledge for risk assessment and control of this important plant pathogen.

## Data availability statement

The original contributions presented in the study are included in the article/**Supplementary Material**. Further inquiries can be directed to the corresponding authors.

## Author contributions

MS: Conceptualization, Investigation, Methodology, Writing – original draft, Data curation. KW: Data curation, Investigation, Methodology, Writing – review & editing. MT: Writing – review & editing. TS: Writing – review & editing. AO: Writing – review & editing. SB: Investigation, Writing – review & editing. LS-R: Investigation, Writing – review & editing, Methodology. RW: Investigation, Methodology, Writing – review & editing, Data curation, Funding acquisition. TS: Methodology, Writing – review & editing, Conceptualization. JS: Conceptualization, Methodology, Funding acquisition, Investigation, Writing – original draft.
