# Supplementary figures and images for: Filling out the gaps – identification of fugralins as products of the PKS2 cluster in Fusarium graminearum
Source: Front Fungal Biol. 2023 Nov 10;4:1264366. doi: 10.3389/ffunb.2023.1264366 (PMC10667903; doi:10.3389/ffunb.2023.1264366)

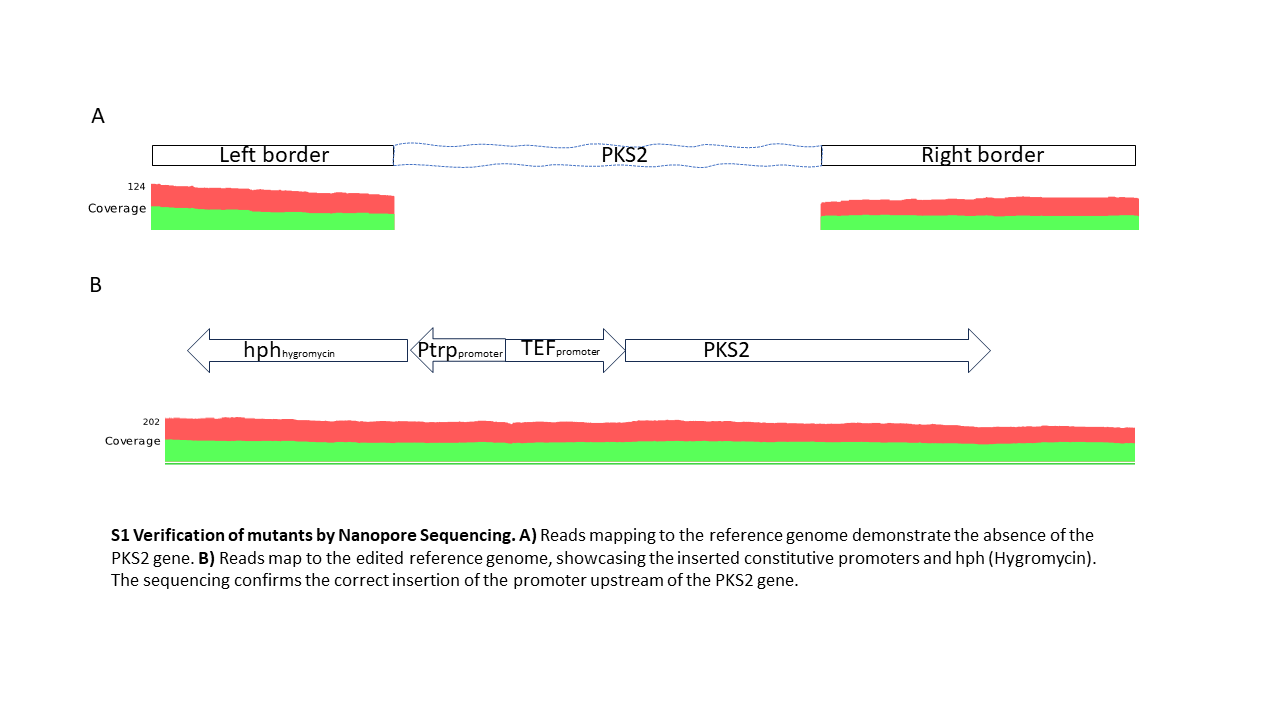

Supplement: Supplementary file 1 [file Image_1.tif]
